# Psychopathology and Somatic Complaints: A Cross-Sectional Study with Portuguese Adults

**DOI:** 10.3390/healthcare9040478

**Published:** 2021-04-17

**Authors:** Joana Proença Becker, Rui Paixão, Manuel João Quartilho

**Affiliations:** 1 Faculty of Psychology and Education Sciences, University of Coimbra, 3000-115 Coimbra, Portugal; rpaixao@fpce.uc.pt; 2 Faculty of Medicine, University of Coimbra, 3000-548 Coimbra, Portugal; mjquarti@ci.uc.pt

**Keywords:** psychosomatic, somatic symptoms, psychopathology, Portuguese population

## Abstract

(1) Background: Functional somatic symptoms (FSS) are physical symptoms that cannot be fully explained by medical diagnosis, injuries, and medication intake. More than the presence of unexplained symptoms, this condition is associated with functional disabilities, psychological distress, increased use of health services, and it has been linked to depressive and anxiety disorders. Recognizing the difficulty of diagnosing individuals with FSS and the impact on public health systems, this study aimed to verify the concomitant incidence of psychopathological symptoms and FSS in Portugal. (2) Methods: For this purpose, 93 psychosomatic outpatients (91.4% women with a mean age of 53.9 years old) and 101 subjects from the general population (74.3% women with 37.8 years old) were evaluated. The survey questionnaire included the 15-item Patient Health Questionnaire, the 20-Item Short Form Survey, the Brief Symptom Inventory, the Depression, Anxiety and Stress Scale, and questions on sociodemographic and clinical characteristics. (3) Results: Increases in FSS severity were correlated with higher rates of depression, anxiety, and stress symptoms. The findings also suggest that increased rates of FSS are associated with lower educational level and female gender. (4) Conclusion: Being aware of the relationship between FSS and psychopathological symptoms and the need to explore psychosocial issues during clinical interviews may favor early detection of these cases. The early detection of mental disorders is essential for individuals’ adherence to treatments, reflecting on healthcare costs.

## 1. Introduction

Functional somatic symptoms (FSS) refer to persistent bodily symptoms that lead individuals to seek health care, and that no organic pathology is found that explains such symptoms [1,2,3,4]. FSS are common in medical practice and have frustrated doctors and patients as it is a diffuse condition, difficult to treat, and that can cause considerable disability [5]. More than the presence of unexplained symptoms, FSS are associated with functional disabilities, psychological distress, increased use of health services, and they have been linked to depressive and anxiety disorders [6,7,8,9].

The impact on public health and the economy has stimulated psychiatric attention and research, especially due to the difficulty of diagnosing individuals with FSS [10,11]. Most of the published research on this field has reported high correlations between FSS and depressive and anxiety disorders [12,13,14,15,16], pointing out as the main reason for the assessment of psychopathological symptoms being included when measuring the severity of somatic symptoms [17,18,19,20]. In addition to the relationship of FSS with mental disorders, studies have shown a significant influence of sociodemographic characteristics on the severity of the condition presented by individuals with FSS [10,21,22]. However, the results on this relationship are controversial. Some studies have found that greater somatic symptoms are associated with lower educational level [13,14,22], while others have found an association with higher educational level [10]; studies have indicated that older individuals have a higher rate of FSS [8], while others have found the opposite [12,13]; and, although studies have presented gender and income as relevant factors [13,22], others have found no relationship between these variables and the severity of FSS [1,11,12]. An explanation for the opposite directions of the studies may be related to the cultural and social characteristics. A study that included seven European countries found that the perception of health differs among countries and that geographic location was positively associated with somatic symptoms, with coming from Lithuania and Portugal being among the risk factors for the development of symptoms [23]. “Thus, somatic presentations can be viewed as expressions of personal suffering inserted in a cultural and social context” [24] (p. 310).

One of the difficulties for a proper diagnosis may be related to individuals underestimating the severity of mental health problems and focusing on physical health, which is recurrent among who those have FSS, requiring the physician to pay attention to the emotional, social, and cultural factors associated with somatic complaints [25]. Therefore, this study is worthwhile in that it draws attention to the possible concomitant incidence of psychopathological symptoms and FSS. This study was aimed to examine the relationship between health conditions (which included health perception, mental health, and physical and social functioning), psychopathological symptoms, sociodemographic characteristics (i.e., gender, age, and educational level), and FSS. According to our observations as professionals working in health services in Portugal, in addition to the findings of studies that have shown the relationship between FSS and this set of factors [8,13,14,23,25], we hypothesized that individuals with FSS would be mostly female, with a lower educational level and older age. We also hypothesized that the severity of FSS would be associated with the presence of psychopathological symptoms, as well as FSS would be negatively correlated with health conditions.

## 2. Materials and Methods

### 2.1. Participants and Procedure

A sample of 93 psychosomatic outpatients (clinical group) and a sample of 101 subjects from the general population were included in this study in order to verify the incidence of psychopathological symptoms in those who have FSS complaints. Since the current population of Portugal is 10 million, a sample of 194 may represent results with a confidence level of 90% and a margin of error of +/−6%.

The main author of this study conducted a cross-sectional survey at the Department of Psychosomatic Medicine of the Coimbra Hospital and University Center (CHUC) throughout 10 months. The outpatients’ psychiatrist informed patients that a doctoral study was taking place at the CHUC and, if they were interested in participating, the psychiatrist introduced them to the researcher (main author), who explained the study. After obtaining participant’s informed consent, the researcher asked them to answer the questionnaire. All patients who consulted the psychiatrist due to psychosomatic conditions were invited to participate in the study. The eligibility criteria for sample selection were outpatients over 18 years old and the providing of written informed consent. Exclusion criteria were severe physical illness and mental conditions that made participation difficult (i.e., when the patient was not able to understand the questions or when he/she presented physical limitations—pain, palsy—that prevented him/her from participating in the study). The psychiatrist considered such criteria before introducing patients to the researcher. The 93 outpatients who met the eligibility criteria were included in this study. The eligibility criteria for the selection of the general population sample were individuals over 18 years old, able to understand and complete the questionnaire, and who provided written informed consent. The exclusion criterion was the presence of mental illness or chronic physical illness, which was verified through a question included in the survey questionnaire. This sample was selected through the snowball sampling method. The main author selected an initial sample that subsequently recruited additional subjects. Thus, 101 individuals from the general population met the eligibility criteria. 

This study was approved by the Ethics Committee of the Faculty of Medicine of the University of Coimbra.

### 2.2. Measures

The survey questionnaire included questions on sociodemographic and clinical characteristics, and the following instruments: 

The Patient Health Questionnaire-15 (PHQ-15) derived from the full Patient Health Questionnaire, inquiring about “15 somatic symptom or symptom clusters that account for more than 90% of the physical complaints reported in the outpatient setting” [19] (p. 259). This self-report questionnaire asks respondents to indicate how much they have been bothered by these symptoms within the past four weeks, and the response format is a 3-point Likert scale: not bothered at all (0), bothered a little (1), and bothered a lot (2). Symptom severity is measured in a scale of 0–30: 0–4 = no minimal; 5–9 = low; 10–15 = medium; and 15–30 = high. PHQ-15 has been widely used as a screening instrument for FSS in different healthcare settings, as well as in scientific research, as it is considered reliable and valid for general and clinical populations (Cronbach’s α = 0.80) [18,19].

The 20-item Short-Form Health Survey (SF-20) was designed to reduce respondent burden while being a comprehensive and psychometrically sound general health survey, and yet, it is short enough to be practical for use in large-scale studies with individuals in clinical settings [26]. SF-20 is an instrument composed of 20 items that represent six concepts of health: physical functioning, which includes items for measuring physical limitations and capacities, mobility, and self-care; role functionality and social functioning, both defined by consequent limitations of health problems; mental health, assessed in terms of psychological distress and well-being; health perceptions, evaluated through the indication of respondents about their health in general; and pain, included to verify physical discomfort. The response format of SF-20 is a Likert scale (from 3 to 6 points). For all measures, scores are transformed linearly to 0–100 scales. The health concepts described by the SF-20 range in score from 0 to 100, with higher scores indicating higher levels of function or better health. Internal consistency reliabilities (Cronbach’s α) of the measured health concepts range from 0.81 to 0.88 [27].

The Brief Symptom Inventory (BSI) allows the evaluation of 9 dimensions (psychoticism, somatization, hostility, obsessions-compulsions, paranoid ideation, anxiety, phobic anxiety, depression, and interpersonal sensitivity). The reliability for the 9 symptom dimensions ranges from 0.62 to 0.80 (Cronbach’s α). The lowest alpha values were observed in the phobic anxiety and psychoticism dimensions. The other dimensions showed values above 0.72, with the somatization dimension having the highest value 0.80. This self-report questionnaire asks respondents to indicate how much they have been bothered by a set of symptoms and feelings over the past week, and the response format is a 5-point Likert scale, ranging from 0 (“nothing”) to 4 (“extremely”). The BSI consists of 53 items and can be applied for the screening of somatization, depression, and anxiety [28,29]. 

The Depression Anxiety and Stress Scale (DASS-21) was designed to measure the emotional state of depression, anxiety, and stress, asking respondents to indicate how much the statements apply to them over the past week. The response format of DASS-21 is a 4-point Likert scale, ranging from 0 (“never”) to 3 (“almost always”) [30]. Scores of each scales of this instrument are calculated by summing the correspondent items, and recommended cut-off scores are as follows: Depression: 0–9 = normal; 10–13 = Mild; 14–20 = Moderate; 21–27 = Severe; 28+ = Extremely Severe. Anxiety: 0–7 = normal; 8–9 = Mild; 10–14 = Moderate; 15–19 = Severe; 20+ = Extremely Severe. Stress: 0–14 = Normal; 15–18 = Mild; 19–25 = Moderate; 26–33 = Severe; 34+ = Extremely Severe. The overall score, which includes all items also presents high consistency (Cronbach’s α = 0.88) [31]. 

### 2.3. Statistical Analyses

The sociodemographic and clinical characteristics of the participants were analyzed using frequency analysis. Correlations between age, educational level, PHQ-15 scores, and all dimension scores measured by the SF-20, BSI, and DASS-21 instruments were performed using the Pearson’s correlation test. In addition, studies on comparability between groups were carried out.

Considering the predictable correlation between the dimensions that measure the presence of psychopathological symptoms (BSI and DASS-21) and the dimensions that measure health conditions (SF-20), a principal component analysis (PCA), selecting direct oblimin rotation, was performed. Then, two factors were extracted: psychopathology and health conditions. Subsequently, hierarchical linear regressions (HLR) were conducted in both groups, separately, to estimate the relationship between a set of possible predictive variables and FSS. The variables extracted in the PCA were selected for these analyses. Since recent studies [2,11,32] have mentioned the need to verify the influence of gender, age, and educational level in the variation of somatic symptom severity, these variables were inserted into the model. The order to insert variables in the model was determined by previous studies on the relationship between somatic symptom severity and psychopathology [11,33,34]. Thus, these analyses included 5 blocks: Block 1: gender; Block 2: age; Block 3: educational level; Block 4: psychopathology; Block 5: health conditions. The “Enter” method was used in all blocks [35]. Although there is a slight deviation from normality (negative asymmetry), the residual values are in accordance with what would be expected. The F-test and residual analysis had shown that the assumptions of normality, homoscedasticity, and linearity to perform HLR were met.

All analyses were performed with IBM SPSS^®^ 22.0 (IBM Corporation, Armonk, NY, USA).

## 3. Results

Both samples were consisted predominantly of women: the clinical group consisted of 91.4% (*n* = 85) of women, and the general population consisted of 74.3% (*n* = 75) of women. The clinical group had a higher mean age (53.9 years old) and lower educational level (M = 9.4 years of schooling) in comparison with the general population, which consisted of participants with a mean age of 37.8 years old and 14 years of schooling. A T-test indicated that the sociodemographic characteristics of the groups were significantly different (*p* < 0.001), including regarding the gender (*p* = 0.001). Cohen’s *d* indicated a large effect size on age (1.49) and educational level (1.07), and a medium effect on gender (0.46). The clinical group consisted of subjects diagnosed with fibromyalgia (75.3%), depressive disorder (51.6%), and/or anxiety disorder (14%). The diagnoses were provided by their psychiatrist, in addition to the questionnaire including this question, which was answered by all participants. The general population reported not having been diagnosed with a mental disorder or other medical condition relevant to this study. The most reported somatic complaints in both groups were back pain, fatigue, pain in the arms, legs, or joints, headaches, and trouble sleeping. Sociodemographic and clinical characteristics are provided in Table S1.

Correlation tests revealed that the severity of somatic symptoms was related to the presence of psychopathological symptoms, as well as to poor health conditions. In the general population, all dimensions measured by BSI and DASS-21 were found to be highly associated with the somatic symptom severity (*r* > 0.35, *p* < 0.001); only phobic anxiety had a lower correlation coefficient (*r* = 0.343, *p* < 0.001). Regarding the dimensions measured by SF-20, health perceptions, role functionality, mental health, and pain were significantly correlated with somatic symptoms (*r* > 0.35, *p* < 0.05) in this sample. In the correlation test performed considering the clinical group, all dimensions measured by BSI and DASS-21 had a high association with the severity of the somatic symptoms (*r* > 0.35, *p* < 0.001). However, the DASS-21 depression dimension and the BSI hostility dimension showed a descriptively lower correlation coefficient (*r* = 0.31, *p* < 0.05). In both the general population and the clinical group, anxiety symptoms had the highest correlation coefficient (*r* = 0.563 and *r* = 0.617, respectively, *p* < 0.001). 

PCA was performed, confirming that a set of variables measured by BSI, DASS-21, and SF-20 could be summarized in a few numbers of factors (KMO = 0.950; Bartlett *p* < 0.001). The extraction method was PCA with an oblique (Direct Oblimin with Kaiser Normalization) rotation. Then, two factors explaining 75.5% of the common variance were extracted in this analysis (Table S2): psychopathology (all dimensions measured by DASS-21 and BSI), which explained 67.64% of the variance (amount of variance = 11.5), and health conditions (the dimensions physical functioning, role functionality, social functioning, mental health, and health perceptions measured by SF-20), which explained 7.87% of the total variance (amount of variance = 1.34). The variable SF-20 pain did not seem to load on any factor and, therefore, it was removed from this analysis. Factor 1 and factor 2 showed good internal consistency, with Cronbach’s α values of 0.90 and 0.82, respectively.

The HLR analysis conducted with the general population group indicated that the model explained 23.8% of the variation in the severity of somatic symptoms (R^2^_a_ = 0.238). The analysis showed that gender had the greatest influence on FSS severity (11.6%), which was followed by educational level (7.8%), and psychopathology (6.8%). The variables age and health conditions had no influence on the model (*p* > 0.05). According to the analysis of variance (ANOVA), the model is valid (*p* < 0.001). The HLR analysis conducted with the clinical group showed that only the variable psychopathology had an influence on FSS severity (ΔR^2^ = 0.216; *p* < 0.001). ANOVA showed that the model is valid after entering this variable into the analysis (*p* = 0.000). The HLR outcomes are provided in Table S3.

## 4. Discussion

This study aimed to explore the association between psychopathology and FSS as well as the potential effects of sociodemographic characteristics on FSS in a sample of Portuguese adults. Initially, we aimed to verify the prevalence of psychopathological symptoms in people with somatic complaints; however, we included the characteristics of gender, age, and educational level in the analysis, since research in the field has pointed out the need to verify the influence of these factors on FSS [2,11,32,34]. The significance of the variables gender and educational level in the general population is in line with the literature (though, see 10, 11, and 12 for opposite results), as well as with the characteristics observed in the clinical group, consisting of a majority of women with lower educational level. According to the literature [32,36,37], women generally report more bodily distress and more frequent somatic symptoms than men, and depressive and anxiety disorders are more prevalent in women as well. Studies on somatic symptoms have mentioned the influence of educational level [10,13,14,22], but the importance of this factor remains unexplored. Although the findings of this study suggest a possible association between these variables (gender and educational level) and the FSS, it must be confirmed. Contrary to what we had predicted, age had no significance in the variation of FSS severity. 

In terms of comorbidities, FSS are associated with psychiatric disorders, with high rates of depressive and anxiety symptoms [4,9], which was corroborated by the current study, which found a significant association between all psychopathological symptoms and the FSS severity, with anxiety symptoms having the highest correlation coefficient in both groups. In addition, a significant correlation (*p* < 0.001) was found between the severity of somatic symptoms and obsessive-compulsive symptoms, psychoticism, and paranoid ideation, which is in accordance with previous studies that point out that personality disorders are associated with severe FSS [38]. 

Despite not investigating the relationship between the social context and FSS, the findings indicated a significant relationship between interpersonal sensitivity, stress, and the severity of somatic symptoms (*p* < 0.001), with high correlation coefficients, especially in the clinical group (*r* > 0.50). This could be explained by the impact of stress on somatic symptoms being mediated by the lack of recognition and social support, which are commonly reported by individuals with FSS. On the other hand, FSS are also related to high negative affect (NA) [39]. Individuals who are high in NA are more likely to report negative affective mood states and dissatisfaction regardless of the situation. Since individuals with FSS tend to be pessimistic, in addition to the high prevalence of personality traits or disorders, having a restricted social network is common [37]. As found by Henningsen et al. [5] (p. 15), characteristics such as “avoidant and anxious attachment patterns and deficits in emotion regulation have also been linked as predisposing factors to the different facets of bodily distress”. 

Studies have shown that FSS have depressive, anxiety, and personality disorders as comorbidities, usually through research conducted in outpatient or inpatient settings [3,12,40]. This study is in line with the previous findings that the increase in psychopathological symptoms is highly related to the severity of somatic symptoms as well as poor perception of health and quality of life. Thus, it corroborates our hypotheses that higher FSS severity would be related to the presence of psychopathological symptoms and poorer health perception.

This study has some limitations. The main limitation concerns the characteristics of the samples and the over-representation of women. Although there was a greater representation of women in both samples, the difference between them (91.4% in the clinical group, and 74.3% in the general population) can be considered a bias, as well as the differences in relation to age and educational level. However, the prevalence of women and the lower educational level in the clinical group can be seen as corroborating that such variables influence on the severity of the FSS. Second, due to the cross-sectional nature of the study, causality could not be inferred. The third limitation could be related to the use of self-report instruments, and the application of the PHQ-15 to infer the presence of FSS. However, although PHQ-15 does not explicitly ask for functional somatic symptoms, “it is highly associated with clinician-rated somatic disorder symptom counts” [32] (p. 30). Despite these limitations, we believe that our study adds relevant information to the body of evidence in this area of research. It constitutes a good starting point for future research on the identification of factors that can influence FSS. Further studies are needed to investigate the causal relationship between psychopathology and FSS and, more than considering sociodemographic characteristics, studying the potential effects of social and cultural contexts can bring innovative data to this field.

## 5. Conclusions

Individuals with FSS have been recurrently associated with high costs for health services, as they demand human resources, time, and several medical tests until the identification of their condition and the referral to the appropriate treatment. The diagnostic problems are usually associated with the individuals’ resistance to reporting emotional issues, the concern about the need to exclude organic disease, or even the physician’s low sensitivity for detecting psychosocial problems [37]. Finally, in addition to bringing unpublished data, this study can draw attention to the need to ensure a faster and more effective detection of pathologies underlying the manifested symptomatology and a more objective intervention tailored to the needs of the individual.

## Supplementary Materials

The following are available online at https://www.mdpi.com/article/10.3390/healthcare9040478/s1, Table S1: Sociodemographic and clinical characteristics, Table S2: Results of PCA: factor loadings of the 2-factor model and communalities, Table S3: Hierarchical linear regression of predictors of FSS severity, with 95% confidence interval (general population).
