# Supplementary material for: Psychopathology and Somatic Complaints: A Cross-Sectional Study with Portuguese Adults
Source: Healthcare (Basel). 2021 Apr 17;9(4):478. doi: 10.3390/healthcare9040478 (PMC8073042; doi:10.3390/healthcare9040478)
Supplement: Supplementary file 1 [file healthcare-09-00478-s001.zip › healthcare-1169897-supplementary.pdf]

Table S1. Sociodemographic and clinical characteristics.

**Baseline characteristic**	General Population (n= 101) **M (SD)**	Clinical Group (n = 93) **M (SD)**
Age	37.8 (12.02)	53.9 (9.46)
Educational level (years of schooling)	14.05 (4.07)	9.44 (4.49)
	**n (%)**	**n (%)**
Gender		
Female	75 (74.3)	85 (91.4)
Male	26 (25.7)	8 (8.6)
Marital status		
Single	49 (48.5)	7 (7.5)
Married/partnered	41 (40.6)	72 (77.4)
Divorced	9 (8.9)	10 (10.8)
Widowed	2 (2.0)	4 (4.3)
Hospitalization (previous 2 years) [a b]	6 (6.7)	21 (22.6)
Reason for hospitalization[a b]		
Clinical assessment	4 (4.4)	2 (2.1)
Surgery	0	13 (14)
Accident	0	2 (2.1)
Pneumonia	0	2 (2.1)
Ischemic stroke	0	1 (1.2)
Suicidal ideation	0	1 (1.2)
Bacteria in the brain	1 (1.1)	0
Most reported somatic complaints		
Back pain	88 (87.1)	86 (92.5)
Fatigue	63 (62.4)	93 (100)
Pain in arms, legs, or joints	65 (64.6)	88 (94.6)
Headaches	57 (56.4)	83 (89.2)
Trouble sleeping	47 (46.5)	82 (88.2)
Substance consumption[a b]		
Tobacco use	17 (18.9)	10 (10.8)
Alcohol use	5 (5.5)	2 (2.2)
Illegal drugs use	2 (2.2)	0

[a] Reflects the number and percentage of participants answering yes" to this question. [b] 11 missing data.

Table S2. Results of PCA: factor loadings of the 2-factor model and communalities

Variable	Pattern Matrix		Structure Matrix		h^2
	F1	F2	F1	F2	
DASS-21 depression	**0.875**	0.018	**0.863**	–0.571	0.744
DASS-21 anxiety	**0.698**	–0.216	**0.843**	–0.686	0.737
DASS-21 stress	**0.881**	–0.011	**0.888**	–0.604	0.789
BSI somatization	**0.465**	–0.517	**0.814**	–0.831	0.808
BSI obsession-compulsion	**0.552**	–0.428	**0.841**	–0.800	0.807
BSI interpersonal sensitivity	**0.900**	0.031	**0.879**	–0.575	0.773
BSI depression	**0.892**	–0.041	**0.919**	–0.641	0.846
BSI hostility	**0.877**	0.107	**0.805**	–0.484	0.654
BSI anxiety	**0.872**	–0.012	**0.881**	–0.600	0.776
BSI phobic anxiety	**0.802**	0.058	**0.763**	–0.483	0.585
BSI paranoid ideation	**0.768**	–0.111	**0.843**	–0.629	0.718
BSI psychoticism	**0.918**	0.046	**0.887**	–0.573	0.788
SF-20 health perception	–0.065	**0.859**	–0.644	**0.903**	0.817
SF-20 physical functioning	0.074	**0.908**	–0.538	**0.858**	0.739
SF-20 role functionality	0.039	**0.953**	–0.603	**0.927**	0.860
SF-20 social functioning	–0.212	**0.648**	–0.648	**0.791**	0.650
SF-20 mental health	–0.615	**0.321**	–0.831	**0.735**	0.747

F1 – Psychopathology (67.64% of Variance); F2 – Health Conditions (7.87% of Variance)

Note: The extraction method was principal component analysis with an oblique (Direct Oblimin with Kaiser Normalization) rotation.

Table S3. Hierarchical linear regression of predictors of FSS severity, with 95% confidence interval.

	General Population			Clinical Group		
	B	SE B	β	B	SE B	β
**Step 1**						
Constant	7.229	0.465		16.212	0.526	
Gender	–3.451	1.027	–0.341***	–0.837	1.794	–0.049
		R^2 = 0.116 (p = 0.001)			R^2 = 0.002 (p = 0.642)	
**Step 2**						
Constant	6.995	1.470		14.269	2.936	
Gender	–3.428	1.042	–0.338***	–0.879	1.801	–0.051
Age	0.006	0.037	0.017	0.036	0.054	0.071
		ΔR^2 = 0.000 (p = 0.867);			ΔR^2 = 0.005 (p = 0.503)	
**Step 3**						
Constant	13.129	2.572		17.226	3.506	
Gender	–4.018	1.022	–0.397***	–0.718	1.791	–0.042
Age	–0.041	0.039	–0.114	0.012	0.056	0.024
Educational level	–0.304	0.107	–0.311**	–0.178	0.117	–0.165
		ΔR^2 = .078 (p = .005)			ΔR^2 = .025 (p = .133)	
**Step 4**						
Constant	13.397	2.478		12.622	3.240	
Gender	–3.550	0.999	–0.350***	–2.003	1.608	–0.117
Age	–0.050	0.038	–0.138	0.009	0.049	0.018
Educational level	–0.338	0.103	–0.346**	–0.137	0.104	–0.128
Psychopathology	3.189	1.155	0.268**	5.627	1.119	0.472***
		ΔR^2 = 0.068 (p = 0.007)			ΔR^2 = 0.216 (p = 0.000)	
**Step 5**						
Constant	12.527	2.529		9.510	3.957	
Gender	–3.118	1.033	–0.308**	–2.068	1.601	–121
Age	–0.045	0.038	–0.125	0.006	0.049	0.011
Educational level	–0.317	0.104	–0.324**	–0.121	0.104	–0.113
Psychopathology	2.959	1.157	0.248*	5.329	1.135	0.447***
Health Conditions	1.416	0.952	0.148	3.507	2.586	0.129
		ΔR^2 = 0.019 (p = 0.141)			ΔR^2 = 0.016 (p = 0.179)	

*$p < 0.05$; **$p < 0.01$; *** $p < 0.001$
